# A Novel HER2 Protein Identification Methodology in Breast Cancer Cells Using Raman Spectroscopy and Raman Imaging: An Analytical Validation Study

**DOI:** 10.1021/acs.jmedchem.4c01591

**Published:** 2024-09-21

**Authors:** Halina Abramczyk, Jakub Maciej Surmacki, Monika Kopeć

**Affiliations:** Laboratory of Laser Molecular Spectroscopy, Department of Chemistry, Institute of Applied Radiation Chemistry, Lodz University of Technology, Wroblewskiego 15, 93-590Lodz, Poland

## Abstract

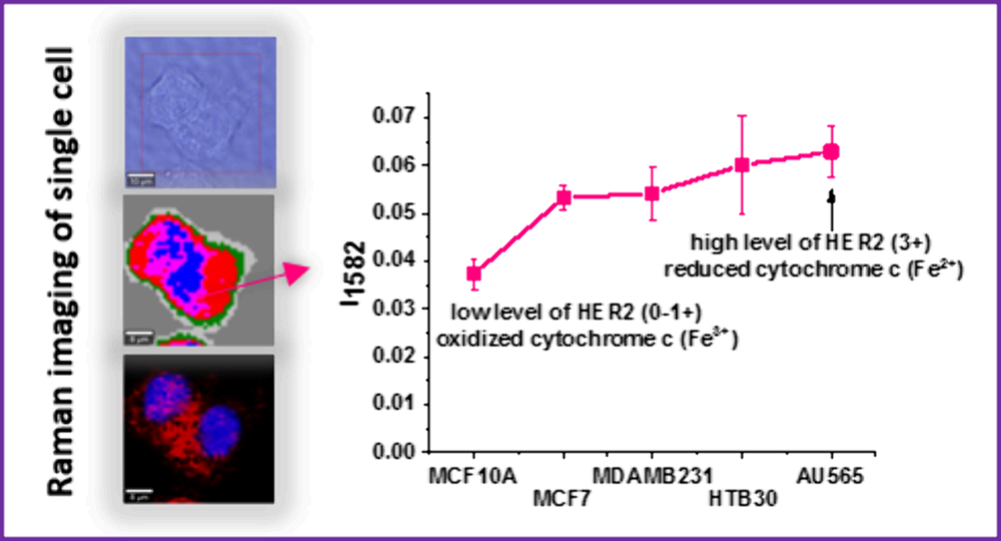

Conventional assays such as immunohistochemistry (IHC) and *in situ* hybridization (ISH) used in clinical procedures for quantification of the human epidermal growth factor receptor-2 (HER2) status in breast cancer have many limitations. In the current study, we have used HER2 expression in a broad range of breast cancer phenotypes to explore the potential utility of a novel immunodetection technique using Raman spectroscopy and Raman imaging combined with artificial intelligence models. The correlations between the Raman method and conventional HER2 testing methodologies (IHC and ISH) have been tested. Raman measurements showed a strong linear correlation (*p* = 0.05, *R*^2^=0,9816) with IHC analysis in the studied breast cell lines: MCF-10A, MCF-7, MDA-MB-231, HTB-30 (SK-BR-3), and AU-565 represent normal, nontumorigenic epithelial cells, triple-positive breast carcinoma, and triple-negative breast cancer cell lines. Analytic testing of Raman spectroscopy and Raman imaging demonstrated that this method may offer advantages over the currently used diagnostic methodologies.

## Introduction

When a virus attacks our body, the entire immune system is alerted through chemical signals of a generated protein. When cancer invades our body, the warning alert is less obvious because cancer cells do not trigger such fierce immune responses as for viruses. The mutated cancer cells are still similar to healthy cells and the immune system does not recognize the distinction between them allowing the cancer cells continuing to grow, divide, and spread throughout the body.

To detect subtle genomic and posttranslational modifications of proteins, DNA, RNA, lipids in cells and tissues, and the cellular environment that occur due to the development of a cancer, we must use proper tools to measure biochemical alterations that are specific for cancer. The characteristic biochemical profiles are called cancer biomarkers that are measured as an indicator of the risk of cancer, occurrence, or patient outcome.^1−5^

An example of a cancer biomarker is the HER2 gene that makes the HER2 protein. Normal tissues have a low amount of HER2 protein, which helps control cell division and growth. Aberrant extra copies of this gene (amplification) may lead to an excess (overexpression) of the HER2 protein, which causes cells to grow more quickly. Overexpressed HER2 is reported in 20% of breast cancer and in some ovarian and gastric cancers.^6−18^

The HER2 protein (also called HER2/neu or ErbB2) is a cellular receptor that is responsible for translating signals from outside the cell into signals within the cell. The routine oncological test for all breast cancers is the determination of HER2 status.

**Figure 1:**
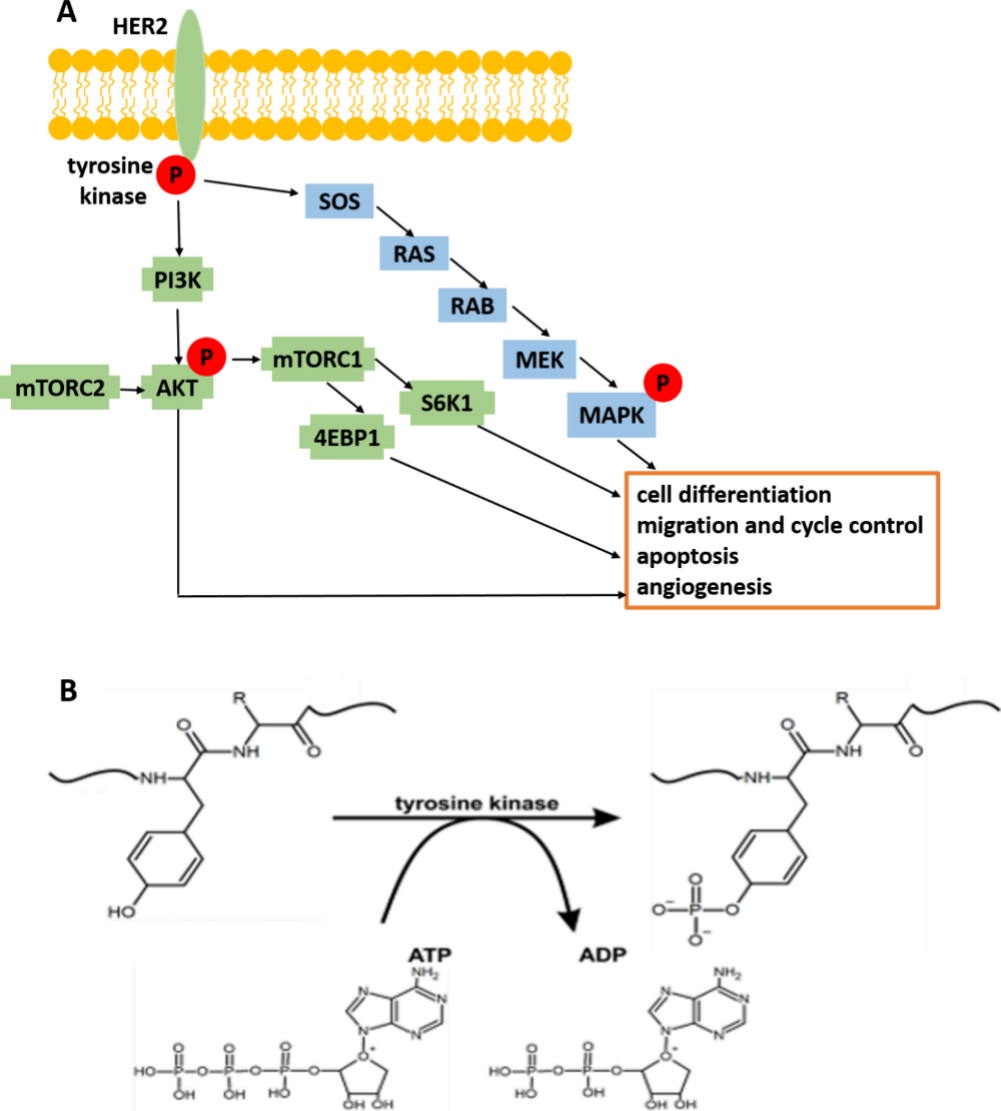
HER2 signaling pathways (panel A) and the effect of phosphorylation catalyzed by tyrosine kinase (panel B).

HER2 belongs to a group of receptor tyrosine kinases (RTKs) and consists of an extracellular domain that includes four subdomains (I–IV),^19^ a single helix transmembrane lipophilic segment, and an intracellular region that contains a tyrosine kinase domain (TKD) and a carboxyl (C−) terminal tail (Figure 1).^20^ RTKs can be activated by ligand-dependent and ligand-independent mechanisms. No ligands for HER2 have yet been identified,^21,22^ and dimerization with any of the other three subdomains is considered to activate HER2.^23^ The dimerization in the extracellular region of HER2 induces intracellular conformational changes that trigger tyrosine kinase activation.^1^

Activated HER2 triggers the phosphorylation of multiple tyrosine (Y) residues at its C-terminus (Figure 1B). Once activated, RTKs play an important role in mediating cell-to-cell communication and controlling a wide range of complex biological functions, including cell growth, motility, differentiation, and metabolism by coupling to PI3K/Akt, Raf/MEK/MAPK, JAK/STAT, Src, PLCγ, and other pathways^24^ (Figure 1A).

In normal cells, the activity of RTKs is governed by mechanisms that keep cell growth under control. Inappropriate activation of HER family receptors, overexpression of HER receptors signal and alterations of RTK pathways results in serious implications in cancer development leading to uncontrolled growth and spread of tumor cells.^25^

Tyrosine phosphorylation is one of the leading areas of biomedical research because of its relation to many human diseases including cancer via the dysregulation of receptor tyrosine kinases.

Protein phosphorylation belongs to the most important covalent post-translational modifications in cell signaling pathways catalyzed by protein kinases, which represents the reversible attachment of a phosphate group to a protein.

Raman spectroscopy and imaging are unique tools that can monitor biochemical profile, localization, and biocomposition alterations in specific organelles of cells as cancer develops.

*In vitro* human cell line models have been widely used to predict the clinical response to mechanisms associated with cancer development. In the current study, five breast cell lines: MCF-10A, MCF-7, MDA-MB-231, HTB-30 (SK-BR-3), and AU-565 were analyzed.

MCF-10A human cells are used as a model for normal, nontumorigenic epithelial human breast cells and represent normal level of HER2. The other studied cell lines represent HER2-positive or HER2-negative breast cancer models. When cells contain higher than normal levels of HER2 they are called HER2-positive. The MCF-7 human cell line has an epithelial-like morphology and represents the human triple-positive breast carcinoma. Triple-positive breast cancer cells use HER2, estrogen receptors, and progesterone receptors to grow. The HER2-positive cancers tend to grow and spread faster than HER2-negative breast cancers but respond more effectively to treatment with drugs that target the HER2 protein. MCF-7 is considered to be a poorly aggressive and noninvasive cell line with low metastatic potential. In contrast, MDA-MB-231 is invasive, poorly differentiated and highly aggressive triple-negative breast cancer cell line without HER2 amplification as well as estrogen receptor (ER) and progesterone receptor (PR) expression.^26,27^ Triple-negative breast cancers are usually more aggressive, more difficult to treat, and more likely to recur than cancers that are hormone-receptor-positive or HER2-positive.

HTB-30 (SK-BR-3) line represents human adenocarcinoma that overexpresses the HER2 and is hormone-independent.^28^ The AU-565 cell line overexpresses the HER2 as well as the HER-3, HER-4, and p53 oncogenes.

Conventional assays used in clinical practice to identify HER2 status in breast cancer include immunohistochemistry (IHC) and in situ hybridization (ISH), both of which have limitations.^29^

In the current study, we studied HER2 expression in the human breast cancer cell lines as a model to explore the potential utility of a novel immunodetection technique based on Raman spectroscopy and Raman imaging combined with methods of artificial intelligence described elsewhere.^30−32^

## Results

Tyrosine phosphorylation is one of the most important post-translational modifications in cancer cells. It is very difficult to monitor these modifications at the genomic level because a large number of proteins (approximately 10,000) encoded by the human genome contain covalently bound phosphate. In proteomic approach, the situation is better because the typical protein kinase can attach phosphates to only 20 proteins.^33^ One of these proteins is cytochrome *c*. It has been proposed that reversible phosphorylation of cytochrome *c* mediated by cell signaling pathways is the primary regulatory mechanism in living species that determines mitochondrial respiration, electron transport chain (ETC) flux, proton gradient ΔΨ_m_, ATP production, and ROS generation. As is well-known, these processes regulate efficiency of the oxidative phosphorylation and is directly related to many human diseases, including cancer, through a lack of energy, ROS production, cytochrome *c* release, and activation of apoptosis.^34^

Because of the importance that phosphorylation has on biological processes in general, a huge emphasis has been placed on understanding the biological role of protein phosphorylation in human diseases. Current approaches of detection strategies include kinase activity assays, immunodetection, phosphoprotein, or phosphopeptide enrichment.^35^

We propose a novel method to monitor HER2 modifications at the molecular level via the unique vibrational signatures of proteins.

The mechanism of tyrosine phosphorylation is presented in Figure 1B. The transfer of the phosphate group to proteins is facilitated by enzymes called tyrosine kinases. The cosubstrate for almost all protein kinases including tyrosine kinase is the donor of phosphate group called adenosine triphosphate (ATP). Tyrosine kinase facilitates the attack of a nucleophilic (−OH) group on tyrosine protein residue on the terminal phosphate group (γ-PO_3_^2–^) on ATP, resulting in the transfer of the phosphate group to tyrosine to form phosphotyrosine and ADP. Protein phosphorylation in a cell is a reversible, dynamic process that is mediated by kinases and phosphatases enzymes, which phosphorylate and dephosphorylate substrates, respectively.^33^

**Figure 2:**
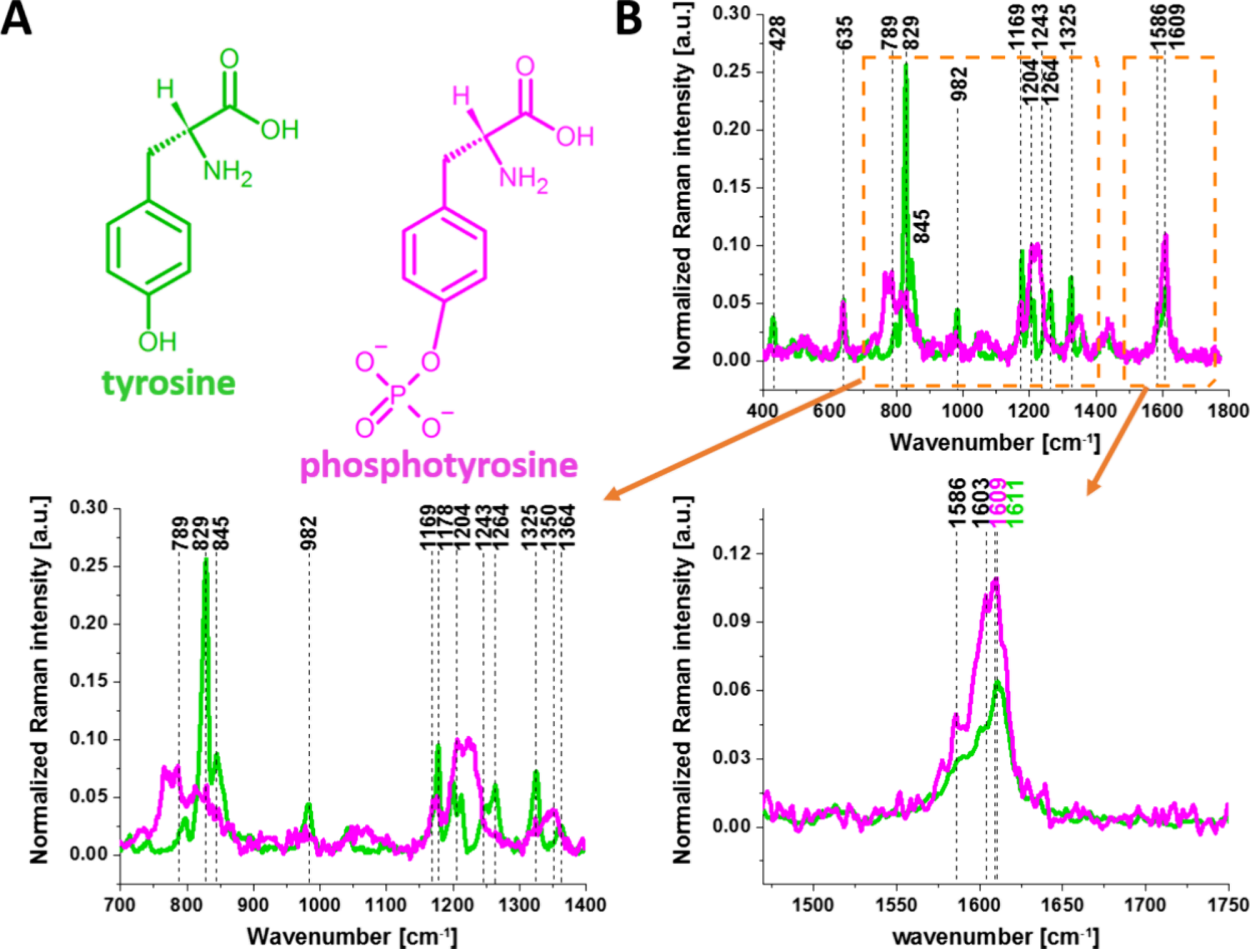
The structural formulas of tyrosine and phosphotyrosine (A) and Raman spectra of tyrosine (green line) and phosphotyrosine (magenta line) (B).^1^

The structural formulas of tyrosine and phosphotyrosine are shown in Figure 2A.

To study the activity of tyrosine kinase presented in Figure 1 by Raman spectroscopy and imaging, let us first concentrate on the Raman vibrational spectra of tyrosine and phosphorylated tyrosine (Figure 2B).

First, we concentrated on the changes in the vibrational landscape that arise in tyrosine upon phosphorylation. The phosphorylation can be monitored by the spectral changes in proteins arising from either phosphate stretching or amide vibrational modes. Recently, comparative structural and vibrational investigations in two different forms of the l-Tyrosine (L-TYR) have been carried out using Raman and IR spectral and DFT methods in the zwitterionic and isolated forms of the molecule.^36^

The Raman spectra of tyrosine and phosphorylated tyrosine are shown in Figure 2B. A detailed inspection into Figure 2B shows that tyrosine phosphorylation induces significant modifications in the Raman vibrational features: (1) an additional Raman peak at 1586 cm^–1^ close to the main peak at 1609 cm^–1^ that corresponds to the ring-O stretching mode^37^ appears in phosphotyrosine; (2) the peak of phosphorylated tyrosine at 1609 cm^–1^ is shifted with respect to that of tyrosine observed at 1611 cm^–1^; (3) collapse into a single band upon tyrosine phosphorylation with a significant intensity decrease of the characteristic doublet (829 cm^–1^, 845 cm^–1^) of tyrosine corresponding to a Fermi resonance between the first overtone of the aromatic out-of-plane ring bend and the aromatic ring breathing fundamental;^38^ (4) the shift of the band at 1264 cm^–1^ corresponding to amide III to 1243 cm^–1^ upon phosphorylation.^39,40^

The band positions of aromatic amino acids are sensitive to the microenvironment and may shift by up to 5 cm^–1^ in the Raman spectra of proteins.^41^ Vibrations of the PO_4_^–^ phosphate group of phosphorylated tyrosine are observed at 1070 cm^–1^ and corresponds to the O–P–O symmetric stretching mode.^38−40,42−44^ The weak band at 1092 cm^–1^ band is due to the antisymmetric O–P–O stretching vibration.^40,43,44^ To summarize, most of the spectral shifts observed upon tyrosine phosphorylation are very similar to those observed in previously reported Raman studies.^37,41,45,46^ The band positions in the Raman spectra of the proteins compared to the reference tyrosine and phosphorylated tyrosine vary by up to only a few cm^–1^ due to their sensitivity to the microenvironment.

**Figure 3:**
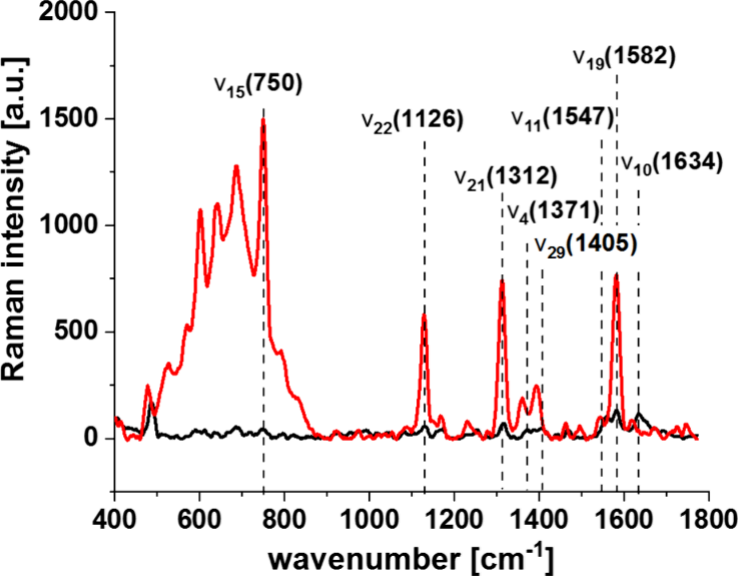
Raman spectra of isolated cytochrome *c* in solution (0.23 mM) dissolved in potassium phosphate buffer, pH 7.4, oxidized ferric Fe^3+^ (black line) and reduced ferrous Fe^2+^ (red line) cytochrome c. Ferrous cytochrome c was prepared by adding a 10-fold excess of reductor ascorbic acid.

One of the important proteins sensitive to phosphorylation is cytochrome c. Figure 3 shows that the Raman spectra of cytochrome *c* in resonance with Q_0_–Q_v_ electronic transitions are dominated by vibrational bands of the asymmetric A_2g_ modes, i.e., 1585 cm ^–1^ (ν_19_) and 1604 cm^–1^ (ν_38_), and from the B_1g_ modes, i.e., 1638 cm ^–1^ (ν_10_) and 1547 cm ^–1^ (ν_11_).^46−48^ The band at 1585 cm^–1^ is primarily due to the methine bridge vibrations via C_α_–C_m_ stretching and C_m_–H bending modes.^47−49^

The ν_15_ vibration at 750 cm^–1^ is associated with the deformation vibrations of the 16 membered inner ring of heme group, ν_4_ vibration at 1371 cm^–1^ involves breathing- like motion of pyrrole ring, ν_29_ vibration at 1405 cm^–1^ represents antisymmetric stretching C_a_-C_b_, the ν_19_ is mainly due to methine bridge stretching C_a_-C_m_ and C_a_-C_b_ vibrations mixed with C_m_-H bending mode with a perpendicular displacement of the C_m_ atom to the plane of the heme group.^48^

Having obtained the reference Raman fingerprint of tyrosine phosphorylation and cytochrome *c*, we focused on the Raman vibrational modifications arising in the proteins due to tyrosine phosphorylation in human normal and cancerous breast *in vitro* cells.

**Figure 4:**
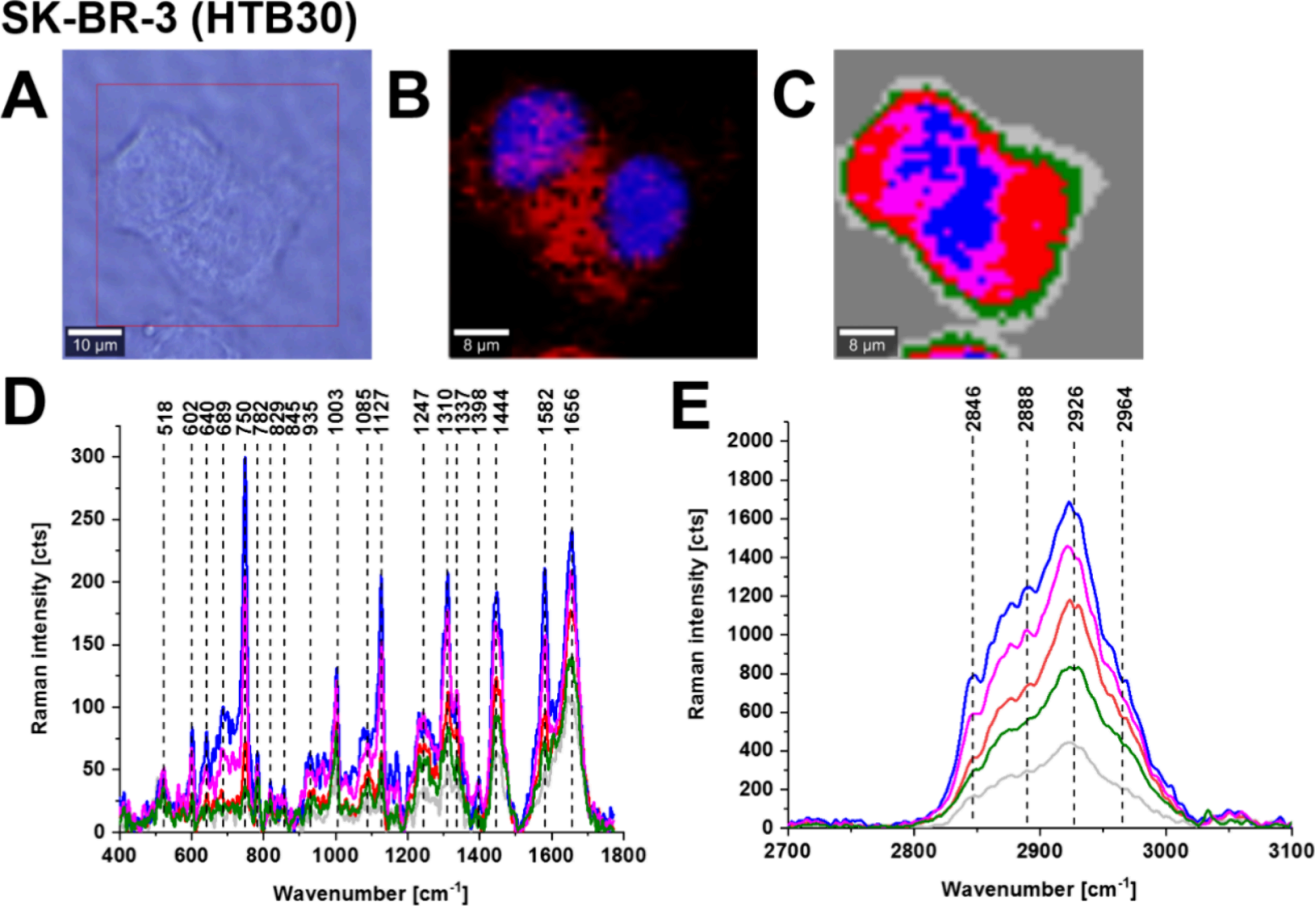
Raman imaging of a typical breast cancer cell (HTB-30). Microscopy images (A), fluorescence image of ER/lipid droplets—red (Oil Red O) (B), fluorescence image of nucleus-blue (Hoechst 33342 (B)), Raman image (C) obtained from the Cluster Analysis (nucleus (red), endoplasmic reticulum (blue), cytoplasm (green), mitochondria (magenta), membrane (light gray)), Raman spectra of the respective organelles (the same color as in Raman image) in the fingerprint region (D) and high-frequency region (E), resolution of Raman images 1 μm, integration time 0.3 s, 10 mW at 532 nm excitation and resolution of 1 μm in fluorescence images, integration time 0.01 s. The colors of spectra correspond to the colors of cluster classes in the Raman maps.

Figure 4 shows the Raman images and Raman spectra for a typical cell of the HTB-30 (SK-BR-3) line. These cells represent human adenocarcinoma and overexpresses the HER2/c-erb-2 gene product.

Figure 4 shows that the Raman spectrum of cancer breast cells of HTB-30 (SK-BR-3) line is dominated by cytochrome *c*. One can see that the vibrations of the heme group in cytochrome *c*: ν_15_ (750 cm^–1^), ν_22_ (1127 cm^–1^), ν_21_ (1310 cm^–1^), and ν_19_ (1582 cm^–1^) are the strongest bands in the cancer breast cell of HTB-30 (SK-BR-3).

**Figure 5:**
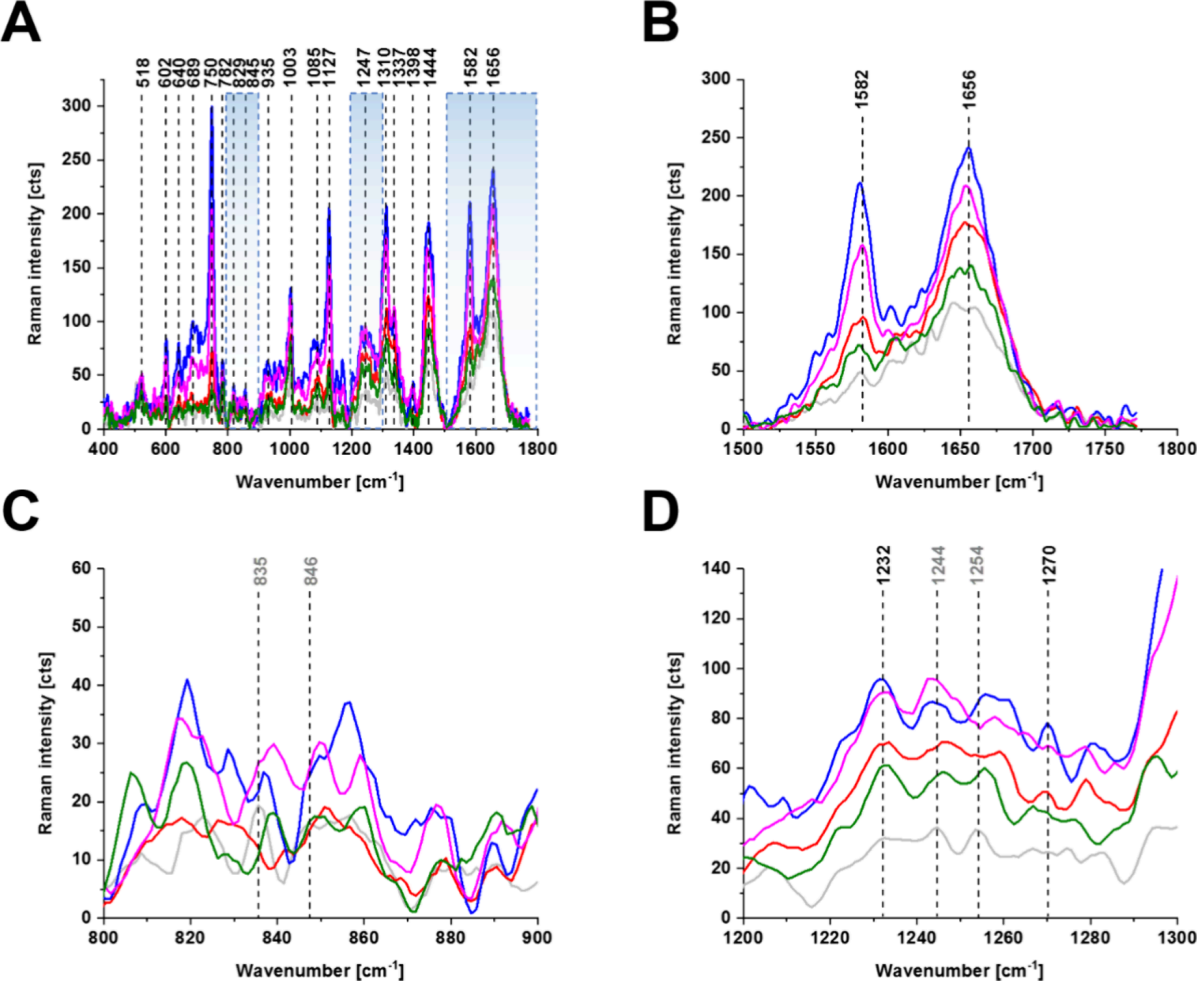
Raman spectra of a typical breast cancer cell (HTB-30) for the cell organelles (nucleus (red), endoplasmic reticulum (blue), cytoplasm (green), mitochondria (magenta), membrane (light gray)). Spectral ranges were 400–1800 cm^–1^ (A), 1500–1800 cm^–1^ (B), 800–900 cm^–1^ (C), and 1200–1300 cm^–1^ (D).

**Figure 6:**
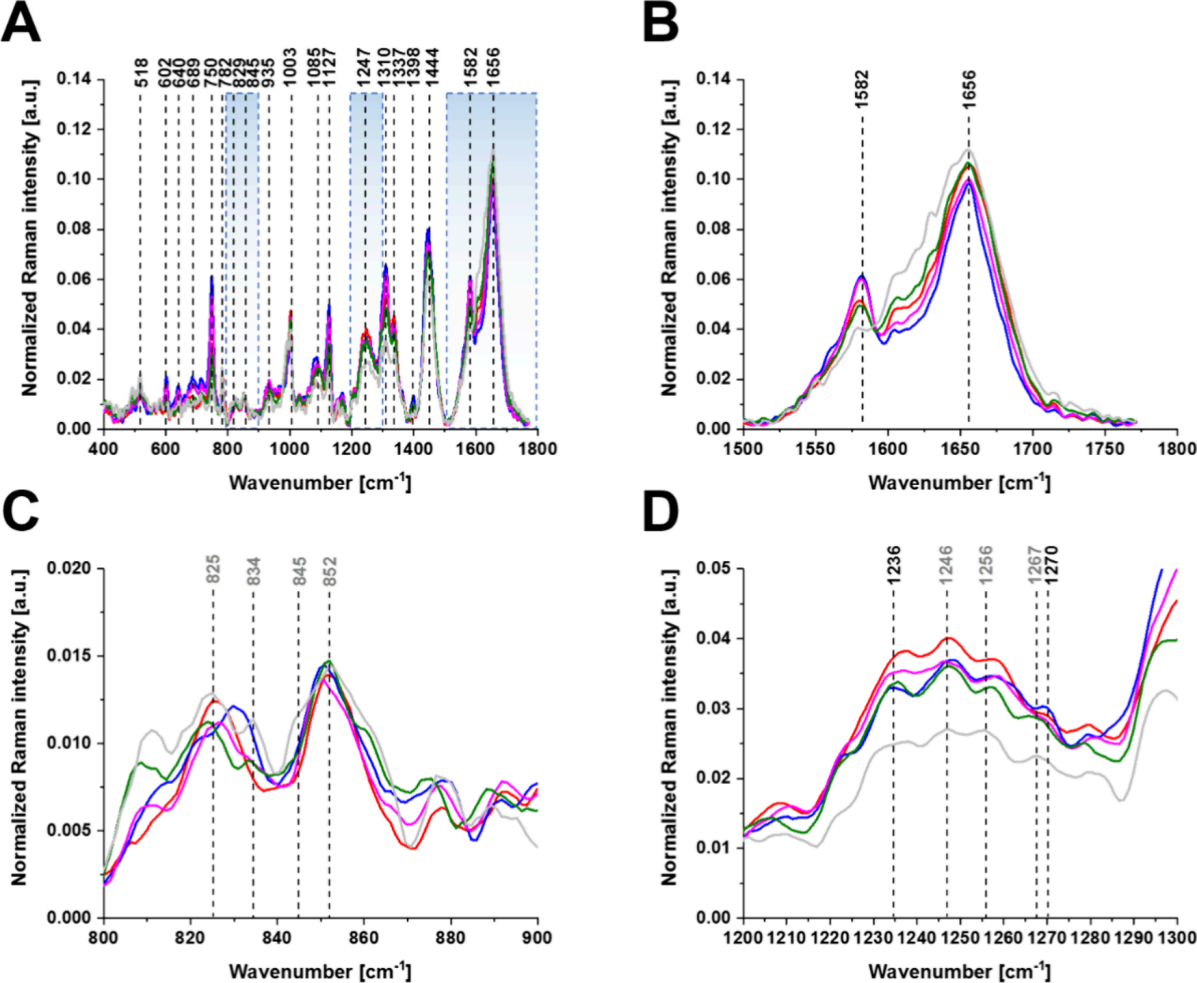
Average normalized Raman spectra of breast cancer cells (HTB-30) for the cell organelles (nucleus (red), endoplasmic reticulum (blue), cytoplasm (green), mitochondria (magenta), membrane (light gray)), average Raman spectra were obtained for the number of cells, *n* = 9. Spectral range of 400–1800 cm^–1^ (A), 1500–1800 cm^–1^(B), 800–900 cm^–1^(C) and 1200–1300 cm^–1^(D).

Detailed inspection into Figures 5 and 6 shows (A) the characteristic doublet (835 cm^–1^, 846 cm^–1^) of tyrosine corresponding to a Fermi resonance between the first overtone of the aromatic out-of-plane ring bend and the aromatic ring breathing fundamental.^48^ The bands do not collapse into a single band as it happens upon tyrosine phosphorylation with a significant intensity decrease as can be seen in Figure 1; (B) the band at 1270 cm^–1^ corresponding to amide III is partially shifted to 1241 cm^–1^ upon phosphorylation.^39,40^ The vibrational features of tyrosine in the cancerous HTB-30 (SK-BR-3) cells illustrate dynamic nature of phosphorylated proteins in a cell^33^ and suggest that the equilibrium between tyrosine and phosphorylated tyrosine is shifted toward nonphosphorylated tyrosine in breast cancer cells.

Figure 5C shows the Raman bands of cytochrome *c* at 1582 cm^–1^ and amide I at 1656 cm^–1^ of a typical breast cancer cell (HTB-30). The equilibrium between the ferric Fe^3+^ and ferrous Fe^2+^ forms is evidently shifted toward ferrous cytochrome *c* in breast cancer cells that overexpress the HER2 proteins.

In the current study, six breast cancer cell lines: MCF-10A, MCF-7, MDA-MB-231, HTB-30 (SK-BR-3), and AU-565 were studied, and the vibrational assignments of the Raman bands at 532 nm laser excitation are shown in Table 1.

**Table 1:** Vibrational Assignments of the Raman Bands Observed in MCF-10A, MCF-7, MDA-MB-231, HTB-30 (SK-BR-3), and AU-565 at 532 nm Laser Excitation

HTB-30 HER-2/neu oncogene adenocarcinoma	MCF-10A control normal	MCF-7 triple-positive poorly aggressive	MDA-MB-231 triple-negative aggressive cancer	AU-565 HER-2/neu oncogene adenocarcinoma	vibrational assignments^50,51^
483	483	483	485	479	glycogen
518	518	518	520	521	phosphatidylinositol
602	602	602	602	601	phosphatidylinositol
640	640	641	638	641	C–S stretching and C–C twisting of proteins-tyrosine C–C twisting mode of tyrosine
689	689	689	687		cytochrome *c*
726	726	726	726		C–S (protein), CH_2_ rocking, adenine
748	749	746	746	748	*c*, *c1* and *b*-types of cytochrome ν_15_ deformation of inner ring of heme group
782	785–787	783	785	785	DNA: O–P–O, cytosine, uracil, thymin Phosphatidylserine
829	829	826	826	823	tyrosine (Fermi resonance of ring fundamental and overtone)
853	845	848	849	852	tyrosine (Fermi resonance of ring fundamental and overtone)
877	877	877	877		C–C–N^+^ symmetric stretching (lipid choline group)
932	932	936	935	932	skeletal C–C, α-helix
1002	1002	1001	1001	1003	phenylalanine
1085	1080–1085	1084	1085	1080 1092	C–C (lipid) Symmetric phosphate stretching vibration of v_3_PO_4_
1127	1124	1126	1125	1126	*c*, *c1*, and *b*-types of cytochrome
1169	1169	1169	1168	1171	tyrosine δ(C–H), tyrosine (protein assignment)
1248	1241	1247	1248	1246	phosphorylated protein amide III
1264	1254	1263	1258/1265	1258	amide III
1311	1306	1306	1302	1308	C-type cytochromes, CH_3_CH_2_ twisting mode of collagen/lipid
1336	1336	1335	1335	1337	cytochrome *b*
1444	1444	1440	1436	1436	cholesterol, CH_2_ deformation
1446	1446	1446	1445	1447	CH_2_ bending mode of proteins and lipids CH_2_ deformation
1456	1456	1456	1459	1460	CH_2_ stretching/CH_3_ asymmetric deformation overlapping asymmetric CH_3_ bending and CH_2_ scissoring (is associated with collagen, and phospholipids)
1578	1576	1578	1578	1576	pyrimidine ring (nucleic acids) and heme protein
1582	1582	1582	1582	1582	cytochrome c ν_19_ methine bridge vibrations via C_α_–C_m_ stretching and C_m_–H bending modes
1602	1600	1602	1602	1602	C=C in-plane bending mode of phenylalanine and tyrosine
1617	1618	1617	1614	1618	C=C phenylalanine, tyrosine
1634	1631	1633	1632	1632	amide I
1647		1647	1648	1647	random coils
1656	1655	1655	1653	1654	amide I
2846	2846	2846	2845	2846	CH_2_ symmetric stretch of lipids
2888	2884	2884	2890	2878	CH_2_ asymmetric stretch of lipids and proteins
2926	2929	2929	2926	2926	symmetric CH_3_ stretch due primarily to protein
2964	2964	2964	2952	2964	v_as_ CH_3_, lipids, fatty cholesterol, and cholesterol ester
3012	3012	3012	3012	3012	unsaturated =CH stretch
3055	3055			3054	aromatic ring =CH, tyrosine

**Figure 7:**
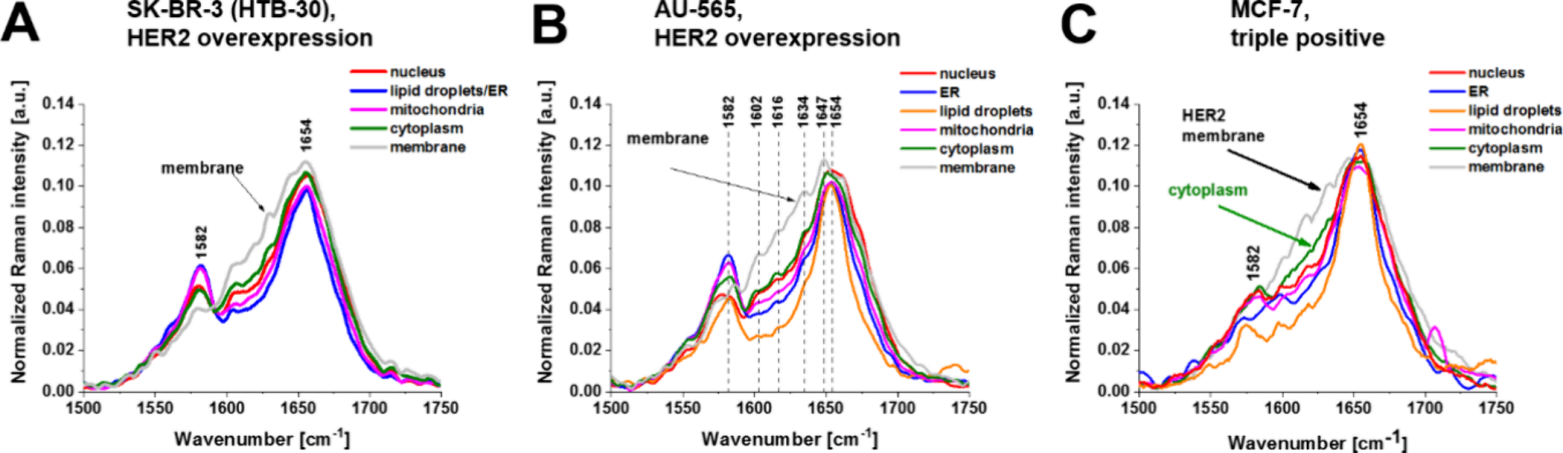
Average normalized Raman spectra of breast cancer cells (HTB-30) (A), AU-565 (B), and MCF-7 (C) for the cell organelles (nucleus (red), endoplasmic reticulum (blue), lipid droplets (orange), cytoplasm (green), mitochondria (magenta), membrane (light gray)).

In Figure 7, we showed average normalized Raman spectra of breast cancer cells (HTB-30) (A), AU-565 (B), and MCF-7 (C) for the cell organelles.

Figure 7 shows average normalized Raman spectra of breast cancer cells (HTB-30) (A), AU-565 (B), and MCF-7 (C) for the cell organelles. These three cancer cell lines overexpress HER2 on the surfaces of cells. Detail inspection into Figure 7 shows that the vibrational profiles of cytochrome *c* at 1582 cm^–1^ and protein Amide I profile at 1654 cm^–1^ in the cell membranes differ spectacularly from the other organelles. Additional bands at 1602, 1618, 1634, and 1647 cm^–1^ appear (Table 1) and most of them correspond to tyrosine vibrations. The presented in Figure 7 cells have HER2 protein expression on the surface of the cell that belongs to a family of receptor tyrosine kinases (RTKs) and consists of an extracellular domain that includes four subdomains (I–IV), a single helix transmembrane lipophilic segment, and an intracellular region that contains a tyrosine kinase domain (TKD).

Our results suggest that we found a novel detection methodology for HER2 protein quantitation in breast cancer cells using Raman spectroscopy and Raman imaging.

The conventional methods of immunohistochemistry give a score of 0 to 3+ that measures the amount of HER2 proteins on the surface of cells in a breast cancer tissue sample. If the score is 0 to 1+, it is considered HER2-negative. If the score is 2+, it is considered borderline. A score of 3+ is considered to be HER2-positive.

To confirm that the HER2 status can be determined by Raman spectroscopy, we extended the list of studied cancer breast cell lines to the normal cells (MCF-10A) and triple-negative aggressive breast cancer (MDA-MB-231).

**Figure 8:**
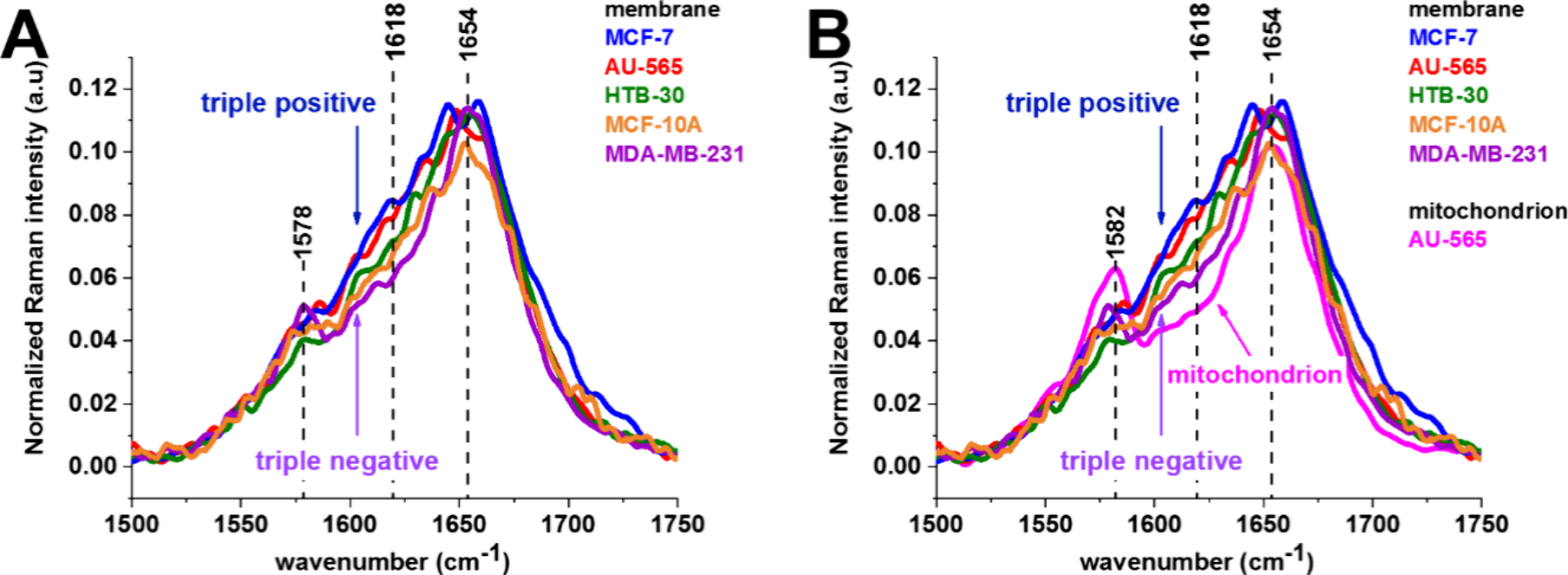
Average normalized Raman spectra of membranes (A, B) and mitochondria (B) in breast cancer cells, triple-positive MCF-7, HTB-30 and AU-565 overexpressing HER2, the normal cells MCF-10A (HER2 at the normal level), and triple-negative aggressive breast cancer MDA-MB-231.

Figure 8A shows average normalized Raman spectra of membranes in breast cancer cells: triple-positive MCF-7, HTB-30, and AU-565 overexpressing HER2, the normal cells (MCF-10A) (HER2 at the normal level), and triple-negative aggressive breast cancer (MDA-MB-231).

Figure 8B shows average normalized Raman spectra of membranes and mitochondria in breast cancer cells (HTB-30) (A), triple-positive HER2MCF-7 (B), HTB-30, and AU-565 (C) overexpressing HER2, the normal cells (MCF-10A) (HER2 at the normal level), and triple -negative aggressive breast cancer (MDA-MB-231).

Figure 8B shows the average normalized Raman spectra of membranes and mitochondria in breast cancer cells, triple-positive HER2MCF-7, HTB-30, and AU-565 (C) overexpressing HER2, the normal cells (MCF-10A) (HER2 at the normal level), and triple-negative aggressive breast cancer (MDA-MB-231). One can see that the highest concentration of cytochrome *c* represented by the vibration at 1584 cm^–1^ is located in mitochondria and supports the results reported recently^1^

**Figure 9:**
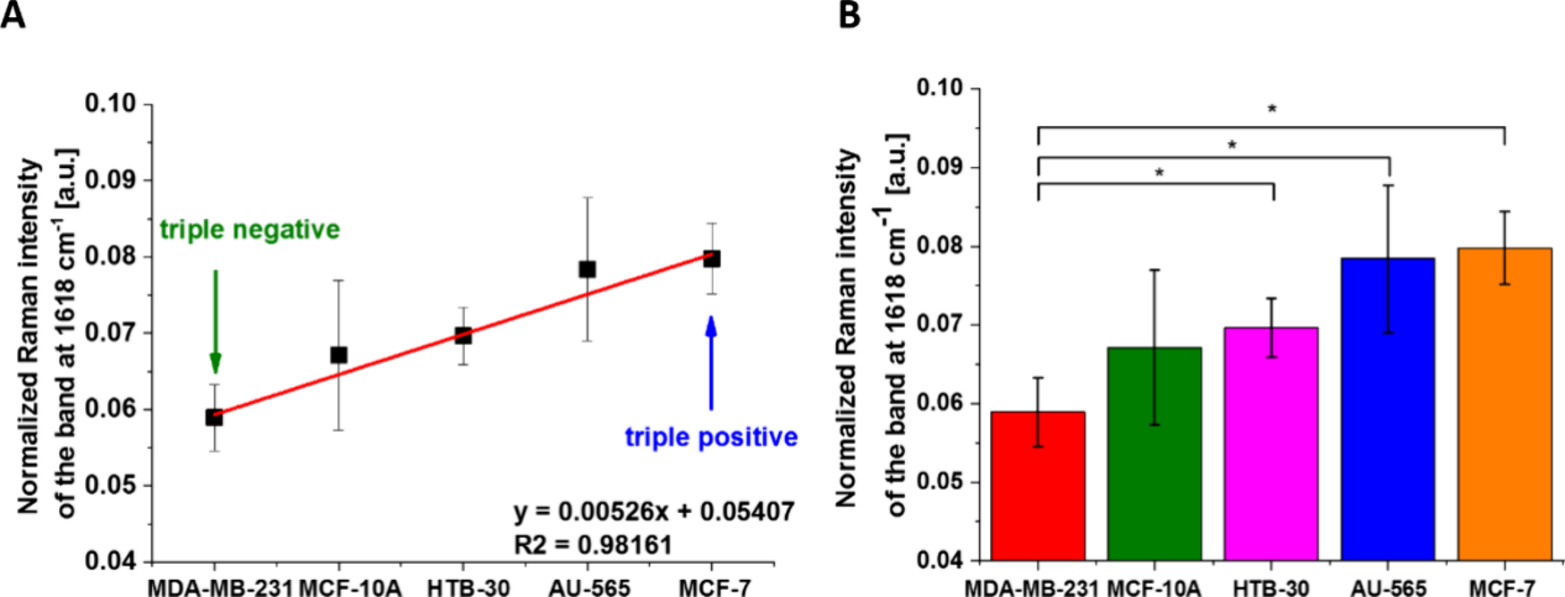
Normalized Raman intensity of the band at 1618 cm^–1^ of breast cancer cells membranes for triple-positive (MCF-7), overexpressing HER2 (HTB-30 and AU-565), the normal cells (MCF-10A) (HER2 at the normal level), and triple-negative aggressive breast cancer (MDA-MB-231). (A) Linear fitting *R*^2^ = 0.98161. (B) Normalized Raman band intensity (1618 cm^–1^) as a function of breast cancer (mean ± SD); the statistically significant results have been marked with an asterisk (*p* < 0.05).

Detailed inspection of Figure 8B shows that the Raman intensity between the cytochrome *c* band at 1582 cm^–1^ and the protein amide I at 1654 cm^–1^ is spectacularly different for mitochondria and membranes of the studied cells. The bands in this region correspond to tyrosine kinase vibrations (Table 1). We have chosen for analysis the Raman intensity at 1618 cm^–1^ representing the tyrosine kinase of HER2. The results for the concentration of tyrosine kinase illustrated by the Raman intensity at 1618 cm^–1^ are presented in Figure 9. One can see that the Raman intensity proportional to concentration of tyrosine kinase receptor at the intracellular side of the membrane for triple-positive MCF-7, HTB-30, and AU-565 overexpressing HER2 is higher than that for the normal cells (MCF-10A) (HER2 at the normal level). In contrast, triple-negative aggressive breast cancer (MDA-MB-231) exhibits a lower Raman signal than the cells (MCF-10A) with HER2 at the normal level.

Figure 9 shows average normalized Raman intensity at 1618 cm^–1^ at membranes (A,B) in breast cancer cells: triple-positive MCF-7 (B), HTB-30, and AU-565 (C) overexpressing HER2, the normal cells (MCF-10A) (HER2 at the normal level) and triple-negative aggressive breast cancer (MDA-MB-231). Figure 9 shows that there is a linear dependence between the normalized Raman intensity of tyrosine vibration at 1618 cm^–1^ on the surface of the normal and cancer cells and the HER2 and EGFR statuses determined by conventional biological methods. It indicates that the HER2 expression evaluated by the Raman method in the cells correlates in an excellent way with the conventional biological methods of HER2 determination.^28^

One can see that Raman spectroscopy shows strong correlations with the results of conventional HER2 testing methodologies by IHC analysis presented in Table S1 for all studied breast cancer cell lines. In contrast, there is no correlation with EGFR receptors. Our results for HER2 receptor kinases in breast cancer are supported by other reports in the literature that the dimerization causes the activation of downstream signaling pathways by phosphorylating the intracellular tyrosine kinase domain*.*^52^

No ligands for HER2 have yet been identified yet.^21,22^ and dimerization with any of the other three subdomains is considered to activate HER2.^23^ The dimerization in the extracellular region of HER2 induces intracellular conformational changes that trigger tyrosine kinase activation.^1^

The intriguing exception is MCF-7 cancer, which does not correlate with HER2 (0–1+) but ER and PR are very high, being 6 and 6, respectively. It was reported that both estrogen and progesterone can interact with membrane receptors.^53^

Breast cancers are categorized into five different subtypes, luminal A/B, HER2-positive (HER^+^), basal-like, claudin-low, and normal breast-like, based on the expression levels of estrogen and progesterone receptors (ER and PR), HER2, cytokeratins 5/6, and claudins 3/4/7.^54−56^

**Figure 10:**
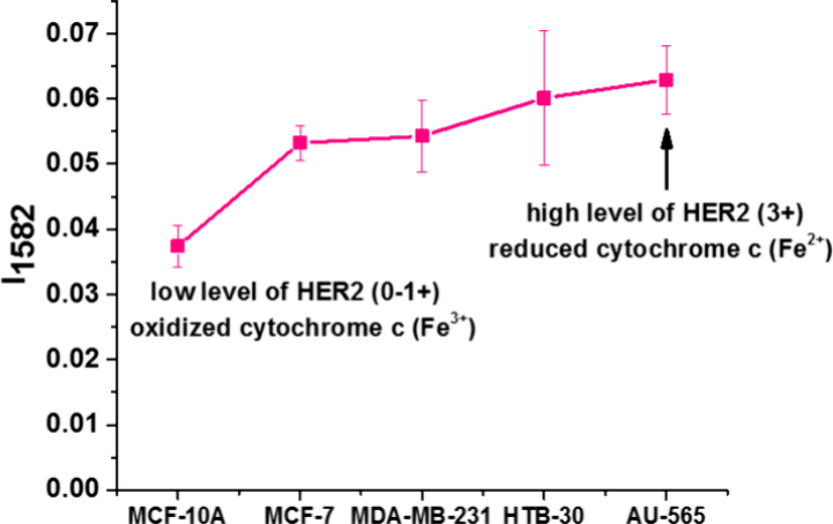
Average normalized Raman intensity of cytochrome *c* at 1582 cm^–1^ in mitochondria of breast cancer cells: triple-positive HER2MCF-7, HTB-30, and AU-565 overexpressing HER2, the normal cells MCF-10A (HER2 at the normal level), and triple-negative aggressive breast cancer MDA-MB-231.

Figure 10 shows the average normalized Raman intensity of cytochrome *c* at 1582 cm^–1^ in mitochondria of breast cancer cells: triple-positive HER2MCF-7 (B), HTB-30, and AU-565 (C) overexpressing HER2, the normal cells MCF-10A (HER2 at the normal level), and triple-negative aggressive breast cancer MDA-MB-231.

One can see that the Raman intensity of cytochrome *c* shows a strong and statistically significant correlation with HER2. The increase in the Raman signal results from a change in the redox status from the oxidized Fe^3+^ to the reduced form Fe^2+^.^4,57^ For cells expressing a high level of HER2, cytochrome *c* in mitochondria exhibits a significant shift toward reduced form Fe^2+^.

## Conclusions

*In vitro* human cell lines are one of the most commonly used preclinical models to examine the biology of cancer tumors to therapy. This paper demonstrates that Raman spectroscopy and Raman imaging have great potential for the quantitative identification of proteins in routine clinical tests. We have shown that the Raman approach demonstrates sensitive and rapid quantitation of HER2 protein in five breast cell lines: MCF-10A, MCF-7, MDA-MB-231, HTB-30 (SK-BR-3), and AU-565. The results obtained from this methodology are highly correlated with routine clinical HER2 testing in *in vitro* human cell line breast cancer models and are very promising for predicting the pathologic response to drug therapy. Raman spectroscopy shows strong correlations with the results of routine HER2 testing methodologies by IHC analysis. The Raman intensity of cytochrome *c* at 1582 cm^–1^ shows a strong and statistically significant correlation with HER2. The increase in the Raman signal results from a shift in the redox status from the oxidized Fe^3+^ to the reduced form Fe^2+^. We showed that for cells expressing a high level of HER2 at the membrane, cytochrome *c* in mitochondria exhibits a significant shift toward reduced form Fe^2+^. The intriguing exception is MCF-7 cancer for which Raman measurements do not correlate with HER2 (0–1+) but correlate with ER and PR receptor expression.

Our findings should be considered preliminary until they will be confirmed by larger than five, well-controlled *in vivo* cell lines and *ex vivo* tissues of patients with HER2-positive and negative breast cancer. Our data suggest that the quantitative Raman measurement of HER2 protein levels may offer an advantage over current clinical testing and will help in proper selection of patients for HER2-targeted therapy. Further studies in *ex vivo* tissues for a large patient cohort are warranted.

## Materials and Methods

### c

Cytochrome (no. C2506, purity SDS-PAGE ≥ 95%), tyrosine (no. T3754, purity HPLC ≥ 98%), and phosphotyrosine (no. P9405, purity HPLC ≥ 98%) were purchased from Merck Life Science.

### Cell Culture Preparation for Raman measurements

Cell lines MCF10A (no. CRL-10317, ATCC), MCF7 (no. HTB-22, ATCC), MDA-MB-231 (no. CRM-HTB-26, ATCC), SK-BR-3 (no. HTB30, ATCC), and AU565 (no. CRL-2351, ATCC) were persuaded from ATCC and cultured according to ATCC protocols. For Raman measurements, cells were seeded onto calcium fluoride windows (CaF_2_, 25 × 2 mm) at a low density of 5 × 10^4^. After 24 h, the CaF_2_ slides with cells were rinsed with phosphate-buffered saline (PBS, SIGMA P-5368, pH 7.4 at 25 °C, c = 0.01 M) to remove any residual medium, and then cells were fixed with 4% formalin solution (neutrally buffered) and kept in PBS (no. 10010023, Gibco) during the experiment. After performing Raman imaging measurements, the cells were treated by 15 min with Hoechst 33342 (25 μL at 1 μg/mL per mL of PBS) and Oil Red O (10 μL of 0.5 mM Oil Red dissolved in 60% isopropanol/dH_2_O per each mL of PBS). Following a PBS wash, the cells were fluorescence imaged using an Alpha 300RSA WITec microscope, with the addition of fresh PBS.

### Raman Spectroscopy and Imaging

Raman measurements of the human breast cell lines were performed with a WITec confocal alpha 300 Raman microscope and a diode laser coupled to the microscope via an optical fiber with a 50 μm diameter core. The 40× objective (NIKON CFI Plan Fluor C ELWD (Extralong Working Distance) 40× : N.A. 0.60, W.D. 3.6–2.8 mm) was used. Before collection of Raman spectra a standard single-point calibration procedure was performed with the reference of Raman band produced by a silicon plate at 520.7 cm^–1^. The Raman spectra were induced by a 532 nm excitation wavelength laser with a power of 10 mW in the focus spot and an integration time of 0.3 s by Andor Newton DU970-UVB-353 CCD camera in enhanced mode (EMCCD). The Raman data analysis was performed by using WITec (WITec Project Plus 4) and OriginPro 2023 software. To construct the Raman maps presented in the paper, we used the Cluster Analysis procedure described in detail elsewhere.^30−32^ The number of clusters used for analysis was 6 (the minimum number of clusters characterized by different average Raman spectra, which describe the organelles in the cell: nucleus, lipid droplets, endoplasmic reticulum (ER), cytoplasm, mitochondria, and cell border). The colors of the cluster images correspond to the colors of the Raman spectra of nucleus (red), lipid droplets (orange), endoplasmic reticulum (blue), cytoplasm (green), mitochondria (magenta), and cell border (light gray).

Numbers of analyzed cells n(MCF-7) = 4, n(HTB-30) = 11, n(AU-565) = 20, n(MDA-MB-231) = 3, and n(MCF-10A) = 3 are the number of Raman spectra of MCF-7, HTB-30, AU-565, MDA-MB-231, and MCF-10A used for averaging 7424, 18125, 8639, 10920, and 5200, respectively.

### ANOVA

The one-way ANOVA test implemented in the OriginPro 2016 software was applied for the statistical analysis of the spectroscopic data. To calculate the value of statistical significance, the Tukey test was used; the asterisk (*) denotes that the differences are statistically significant, *p*-value of ≤0.05.

## Data Availability

The raw data underlying the results presented in the study are available from Lodz University of Technology Institutional Data Access. Request for access to those data should be addressed to the corresponding author.

## Supporting Information Available

Supplementary data for the results of IHC analysis for breast cancer cell lines (Table S1) (PDF)
Molecular formula strings (CSV)

## Abbreviations

ADP: adenosine 5′-diphosphate
AKT: adenosine 5′-triphosphate
a.u.: arbitrary units
ATCC: American Type Culture Collection
ATP: adenosine triphosphate
CaF_2_: calcium fluoride
CCD: charge-coupled device
cm^–1^: 1/centimeter
cts: counts
DNA: DNA
EGFR: epidermal growth factor receptor
EMCCD: enhanced mode CCD
ELWD: extra-long working distance
ER: estrogen receptor
ErbB2: Erb-b2 receptor tyrosine kinase 2
HER2/c-erb-2: human epidermal growth factor type II receptor HER2
ETC: electron transport chain
HER^+^: human epidermal growth factor receptor-2 positive
HER2: human epidermal growth factor receptor-2
HER-3: human epidermal growth factor receptor 3
HER-4: human epidermal growth factor receptor 4
HPLC: high-performance liquid chromatography
IHC: immunohistochemistry
ISH: *in situ* hybridization
JAK: Janus kinase
MAPK: mitogen-activated protein kinase
MEK: mitogen-activated protein (serine/tyrosine/threonine) kinase
μm: micrometer(s)
mm: millimeter(s)
mTORC1: mammalian target of rapamycin complex 1
mTORC2: mammalian target of rapamycin complex 2
n: number
N.A.: numerical aperture
nm: nanometer(s)
p: probability value
p53: tumor protein P53
PBS: phosphate-buffered saline
PI3K/Akt: phosphatidylinositol 3-kinase/protein kinase B
PLCγ: phospholipase Cγ
PR: progesterone receptor
R^2^: coefficient of determination
Raf: rapidly accelerated fibrosarcoma
RAB: Ras-associated binding protein
RAS: rat sarcoma viral oncogene homologue
RNA: ribonucleic acid
ROS: reactive oxygen species
RTKs: receptor tyrosine kinases
TKD: tyrosine kinase domain
SDS-PAGE: sodium dodecyl-sulfate polyacrylamide gel electrophoresis
SOS: Son-of-sevenless
Src: Proto-oncogene tyrosine-protein kinase
STAT: signal transducers and activators of transcription
S6K1: S6 Kinase 1
W.D.: working distance
4EBP1: 4E binding protein 1
