# Supplementary material for: A Novel HER2 Protein Identification Methodology in Breast Cancer Cells Using Raman Spectroscopy and Raman Imaging: An Analytical Validation Study
Source: J Med Chem. 2024 Sep 21;67(19):17629–39. doi: 10.1021/acs.jmedchem.4c01591 (PMC11472312; doi:10.1021/acs.jmedchem.4c01591)
Supplement: Supplementary file 1 — jm4c01591_si_001.pdf [file jm4c01591_si_001.pdf]

Supplementary information

A novel HER2 protein identification methodology in breast cancer cells using Raman spectroscopy and Raman imaging: an analytical validation study

Halina Abramczyk[1,*], Jakub Maciej Surmacki[1], Monika Kopeć[1]

[1]*Laboratory of Laser Molecular Spectroscopy, Department of Chemistry, Institute of Applied Radiation Chemistry, Lodz University of Technology, Wroblewskiego 15, 93-590 Lodz, Poland*
[*]*Author to whom correspondence should be addressed (halina.abramczyk@p.lodz.pl)*

Table S1. The results of IHC analysis for breast cancer cell lines.[1]

Cell line	ER	PR	HER2	CK5/6	EGFR	Ki-67	AR	Subtype
MDA-MB-231	0	0	0–1+	−	1+	100%	8	Basal
MCF-10A	0	0	0–1+	+	2+	30%	0	Basal
SK-BR-3 (HTB-30)	0	0	3+	−	2+	20%	8	HER2
AU-565	0	0	3+	−	1+	95%	7	HER2
MCF-7	6	6	0–1+	−	1+	90%	7	Luminal A

1. Subik K, Lee J-F, Baxter L, et al. The Expression Patterns of ER, PR, HER2, CK5/6, EGFR, Ki-67 and AR by Immunohistochemical Analysis in Breast Cancer Cell Lines. *Breast Cancer (Auckl)*. 2010;4:35-41.
